# Effects of Oral Drugs on Coronary Microvascular Function in Patients Without Significant Stenosis of Epicardial Coronary Arteries: A Systematic Review and Meta-Analysis of Coronary Flow Reserve

**DOI:** 10.3389/fcvm.2020.580419

**Published:** 2020-10-30

**Authors:** Jingwen Yong, Jinfan Tian, Xueyao Yang, Haoran Xing, Yi He, Xiantao Song

**Affiliations:** ^1^Department of Cardiology, Beijing Anzhen Hospital, Capital Medical University, Beijing, China; ^2^Department of Radiology, Beijing Friendship Hospital, Capital Medical University, Beijing, China

**Keywords:** oral drug, coronary microvascular, microvascular function, coronary flow reserve (CFR), therapy

## Abstract

**Objective:** This study aims to investigate the impact of cardiovascular medications on the coronary flow reserve (CFR) in patients without obstructive coronary artery disease (CAD).

**Methods:** We searched PubMed, EMBASE, and Cochrane databases from inception to 15 November 2019. Studies were included if they reported CFR from baseline to follow-up after oral drug therapy of patients without obstructive CAD. Data was pooled using random-effects modeling. The primary outcome was change in CFR from baseline to follow-up after oral drug therapy.

**Results:** A total of 46 studies including 845 subjects were included in this study. Relative to baseline, the CFR was improved by angiotensin-converting enzymes (ACEIs), aldosterone receptor antagonists (ARBs) [standard mean difference (SMD): 1.12; 95% CI: 0.77–1.47], and statins treatments (SMD: 0.61; 95%CI: 0.36–0.85). Six to 12 months of calcium channel blocker (CCB) treatments improved CFR (SMD: 1.04; 95% CI: 0.51–1.58). Beta-blocker (SMD: 0.24; 95% CI: −0.39–0.88) and ranolazine treatment (SMD: 0.31; 95% CI: −0.39–1.01) were not associated with improved CFR.

**Conclusions:** Therapy with ACEIs, ARBs, and statins was associated with improved CFR in patients with confirmed or suspicious CMD. CCBs also improved CFR among patients followed for 6–12 months. Beta-blocker and ranolazine had no impact on CFR.

## Introduction

Patients with angina symptoms without obstructive coronary artery disease (CAD) have been difficult to diagnose and treat. Up to 50–65% of these patients are considered to have coronary microvascular dysfunction (CMD) (1–5), which is associated with diastolic heart failure (6–8). Over the past 20 years, a large number of studies have used invasive and non-invasive imaging techniques to assess coronary microvascular function (9–12) and have increased our understanding of CMD and microvascular ischemia. CMD is a broad term covering four main types and several endotypes, which could be overlap (13–15).

However, there are still no clear guidelines for CMD treatment (16). Although the antihypertensives are not intended for CMD, the recent studies showed that ACEIs, ARBs, and CCBs are potentially useful for improving CRF. Moreover, data on the effectiveness of CMD medications remain scarce. Most studies on this have inconsistent results. Medications including ACEI, statins, and beta-blockers may be used to treat CMD under the current European Society of Cardiology position paper on CMD (17). Several reviews suggest that exercise, controlling risk factors, and medications such as angiotensin-converting enzyme inhibitors (ACEIs), aldosterone receptor antagonists (ARBs), and statins may be effective first-line treatments, and medications including nicorandil and ranolazine can be effective second-line treatments (8, 14, 18, 19).

Statins and ACEIs can improve endothelial dysfunction in patients with hypertension and are also first-line treatments for patients with CMD (20, 21). Ranolazine and ivabradine have been shown to attenuate angina symptoms and improve coronary microvascular function (22–24). Nicorandil is a nitrate and adenosine triphosphate-sensitive potassium channel agonist that can improve peak exercise performance but does not significantly improve the index of microcirculatory resistance (IMR) and ST-induced changes in exercise (22, 25). The proposed treatment algorithm for CMD patients has been summarized by Crea et al. (14). For all patients, efforts should be made to reduce controllable risk factors. Additional traditional and non-traditional anti-ischemic medications are recommended if symptoms are not well-controlled with first-line treatments. Patients with suspected enhanced pain perception should receive pain modulators. For patients with refractory symptoms that significantly restrict the quality of life, treatments such as enhanced external counter pulsation, spinal cord stimulation, and cognitive behavioral therapy should also be considered.

This review focuses on the effects of oral medical therapy on coronary flow reserve (CFR) outcomes in patients with non-obstructed coronary arteries and provides additional evidence to guide physicians in the selection of the optimal pharmaceutical treatment for patients with CMD.

## Methods

### Search Strategy

This study meticulously followed the Meta-analysis of Observational Studies in Epidemiology (MOOSE) guidelines. PubMed, EMBASE, and the Cochrane Central Register of Controlled Trials were searched for relevant publications through November 15, 2019. The search strategies were performed using the following MeSH terms and keywords: coronary microvascular dysfunction, coronary microvessel dysfunction, microcirculation, CFR, coronary flow reserve, coronary flow, treatment, management, therapy, oral drug, and pharmacotherapy (Supplementary Table 1).

We also manually screened the reference lists of included manuscripts to identify any relevant studies not identified by the initial search. The search was restricted to peer-reviewed studies on adults. This systematic review protocol has been registered with the PROSPERO (CRD42020158659).

### Selection Criteria

Two investigators (JWY and TJF) independently selected studies based on the study eligibility criteria. All randomized and non-randomized studies were considered to be eligible if they (1) included patients without significant stenosis of the epicardial coronary artery determined by invasive angiography or CT coronary angiography or other methods; (2) included patients diagnosed with CMD or CAD [acute, stable coronary syndrome; receiving percutaneous coronary intervention (PCI), or coronary artery bypass grafting (CABG)] or having at least one risk factor for coronary heart disease (such as hypertension, hypercholesterol, and diabetes mellitus); (3) included patients receiving CFR testing to evaluate coronary microvascular function before and after treatment; (4) included patients taking one or more of the following oral drugs: statins, ATP-sensitive potassium channel openers, ranolazine, ivabradine, nitrate, CCBs, beta-blockers, ACEIs, ARBs, or trimetazidine; (5) included patients followed up for at least 3 weeks; and (6) were published in English.

**Figure 1:**
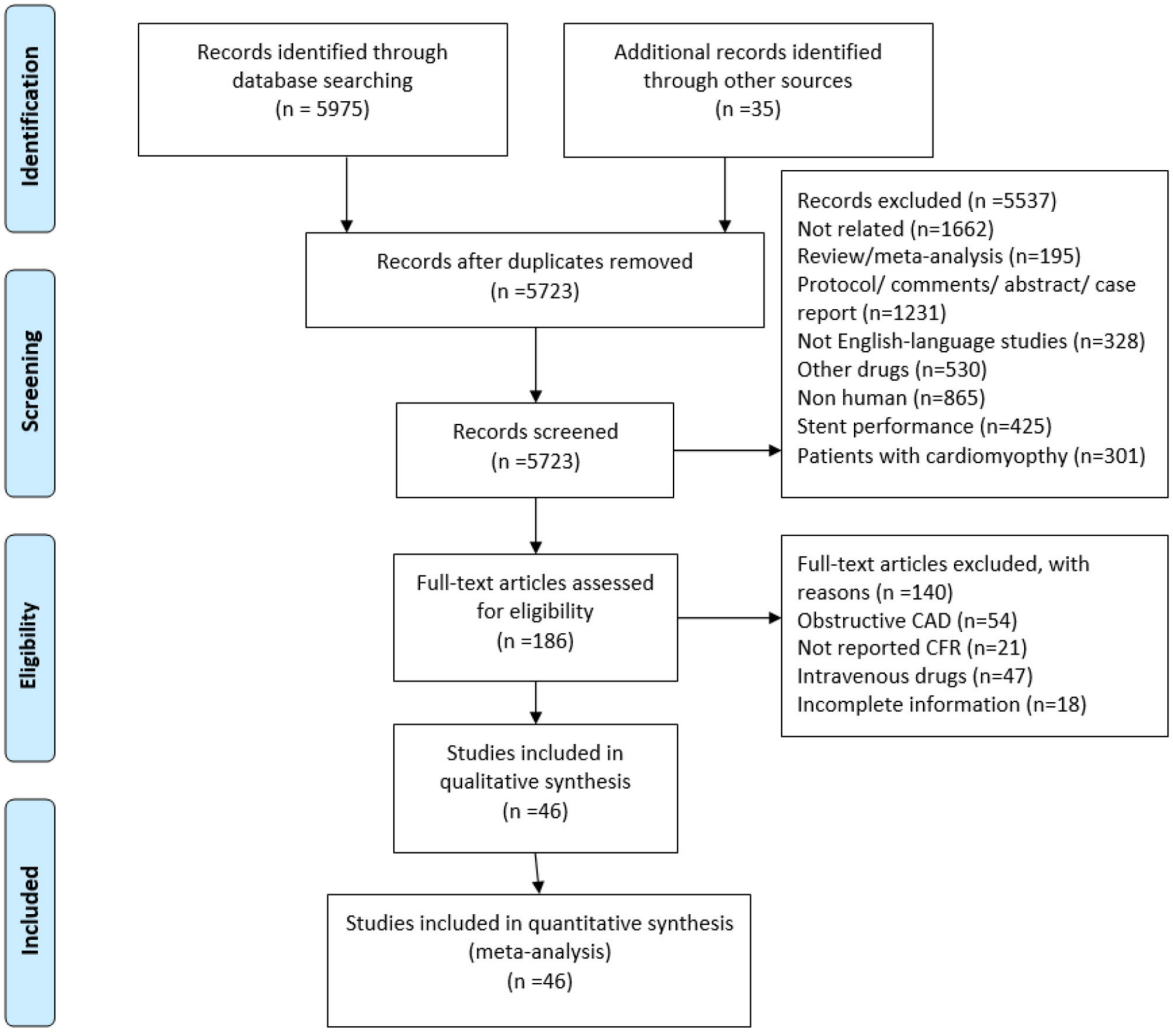
Flowchart of study selection. CMVD, coronary microvascular dysfunction; CFR, coronary flow reserve.

Two researchers independently selected studies based on the study inclusion criteria. Both investigators reviewed the full-text manuscripts of identified articles to assess whether studies met eligibility criteria. We excluded case reports, case series, review, meta-analysis, protocol, comments, abstract, and non-English language studies. Reasons for exclusion during full-text screening included inappropriate population, inappropriate outcome, and insufficient information for outcome assessments, among others. The third opinion (XTS) was consulted to resolve screening discrepancies between the two investigators. The search and screening process is outlined in Figure 1.

### Data Extraction

For included studies, two investigators (JWY and JFT) extracted the data independently. The primary outcome was CFR before and after oral drug therapy. Extracted information included journal, first author name, publication year, population, clinical characteristics at baseline, and patient CFR data before and after treatment. Publication authors were contacted by email when study source data were unclear or to acquire additional data. The third opinion (XTS) was consulted to resolved any data extraction discrepancies between the two investigators.

### Statistical Analysis

Summary results for CFR outcome data are presented as standard mean differences (SMDs) with 95% CI. The *I*^2^ test was applied to assess heterogeneity between studies, where *I*^2^ < 25% was regarded as no heterogeneity; 25–50%, moderate heterogeneity; and >50%, severe heterogeneity. We calculated pool estimates of the means and standard deviations (SD) of pre-CFR and post-CFR between different drug groups using a random-effects model (Der Simonian and Laird method) to account for uncertainty associated with interstudy variabilities in drug effects. Publication bias was assessed using Egger's linear regression test and visual inspections of funnel plots. Analysis was performed using Stata 11.0 (Stata Corp, College Station, TX) and Review Manager, Version 5.3 (Cochrane Collaboration, Oxford, United Kingdom). A two-tailed *P* < 0.05 was considered statistically significant.

## Results

### Study Selection and Characteristics

A total of 5,723 references were identified from database search analyses. Of these, 5,537 were excluded during title and abstract level screening (Figure 1). Of the remaining 186 studies, 140 were excluded for the following reasons: obstructive CAD (*n* = 54), unclear or missing CFR data (*n* = 21); use of intravenous drugs (*n* = 47); and incomplete information (*n* = 18). Forty-six of the remaining studies reported CFR data and did not meet any other exclusion criteria, of which 28 were randomized controlled trials and 18 were non-randomized studies. The study characteristics are presented in Table 1, and the clinical characteristics of patients are presented in Supplementary Table 2. A total of 845 patients, ranging from 8 to 55 participants per trial, were ultimately included who received coronary microvascular function assessments before and after administration of oral medications. CFR is feasible for coronary microvascular function evaluation (1), and we therefore collected CFR data as an indicator of coronary microvascular function. At present, there is no uniform gold standard for CFR detection methods. Methods for measuring CFR included intracoronary (IC) Doppler flow wire (*n* = 6), cardiac magnetic resonance imaging (CMRI) (*n* = 2), positron emission tomography (PET) (*n* = 11), and Doppler echocardiography (DE) (*n* = 27). Methods for obtaining stenosis of epicardial coronary artery included invasive angiography (*n* = 21), CT coronary angiography (*n* = 6), medical history (*n* = 8), and DE and treadmill exercise test (*n* = 11). Follow-up duration varied from 0.75 to 12 months.

**Table 1:** Study characteristics.

**Author**	**Diagnosis**	**Method for CAD**	**Method for CFR**	**Drugs**	**Dose**	**Follow-up months**	***N***	**Pre-CFR**		**Post-CFR**	
								**Mean SD**		**Mean SD**	
**RANDOMIZED**											
Golino et al. (26)	After PCI, SCAD	Invasive angiogram	DE	Ranolazine	750 mg/day	0.75	8	1.33	0.16	1.39	0.29
Safdar et al. (27)	CMD	CTA	PET	Ranolazine	1,000–2,000 mg/day	1	21	1.6	0.3	1.9	0.4
Villano et al. (23)	CMD	Invasive angiogram	DE	Ranolazine	750 mg/day	1	15	1.99	0.6	1.86	0.5
Zhang et al. (28)	Cardiac syndrome X	Invasive angiogram	DE	Diltiazem	90 mg/day	3.25	22	2.19	0.58	2.5	0.72
				Fluvastatin	40 mg/day	3.25	22	2.02	0.45	2.63	0.62
Pauly et al. (9)	CMD	Invasive angiogram	IC Doppler	Quinapril	40–80 mg/day	4	29	2.52	0.36	2.77	0.5
Iino et al. (29)	After PCI in RCA, patients without stenosis in LAD	Invasive angiogram	IC Doppler	Candesartan	4–8 mg/day	6.5	14	1.99	0.2	3.37	0.27
Chen et al. (21)	Cardiac syndrome X	Invasive angiogram	IC Doppler	Enalapril	10 mg/day	2	10	3.26	0.56	4.01	0.65
Toyama et al. (30)	HT	Medical history	CMRI	Olmesartan	10–40 mg/day	6.5	10	1.9	1	3.1	1.1
				Amlodipin	2.5–10 mg/day	6.5	10	2.2	0.8	2.4	0.9
Kamezaki et al. (31)	HT	Clinical history and treadmill exercise test	DE	Valsartan	40–80 mg/day	1.5	8	2.34	0.38	3.04	1.09
				Nifedipine	20–40 mg/day	1.5	8	2.72	0.22	2.41	0.4
Parodi et al. (32)	HT	Invasive angiogram	PET	Enalapril	10–40 mg/day	6.5	10	2.42	0.72	2.37	0.59
				Verapamil	240–480 mg/day	6.5	10	2.74	0.8	3.73	1.79
Hinoi et al. (33)	HT	Medical history	DE	Telmisartan	40 mg/day	5	20	2.4	0.4	2.9	0.4
				Nifedipine	20 mg/day	5	20	2.5	0.3	2.5	0.3
Xiaozhen et al. (34)	HT&LVH	Invasive angiogram	DE	Carvedilol	10 mg/day	6.5	28	2.31	0.31	3.16	0.67
				Metoprolol	50 mg/day	6.5	29	2.32	0.29	2.46	0.58
Gullu et al. (35)	HT	Medical history	DE	Nebivolol	5 mg/day	2	30	2.45	0.48	2.56	0.52
				Atenolol	50 mg/day	2	30	2.46	0.44	2.21	0.4
Buus et al. (36)	HT	Medical history	PET	Perindopril	4–8 mg/day	12	15	2.39	0.17	2.64	0.17
				Atenolol	50–100 mg/day	12	15	2.31	0.16	2.09	0.19
Yokoyama et al. (37)	HC	Echocardiography and treadmill exercise test	PET	Simvastatin	5–10 mg/day	10	22	2.36	0.67	3.18	1.22
				Pravastatin	10–20 mg/day	10	22	2.21	0.72	2.32	0.64
Lario et al. (38)	HC	CTA	DE	Atorvastatin	40–80 mg/day	3	16	2.78	0.71	3.43	0.66
Kawata et al. (39)	DM	Echocardiography and treadmill exercise test	DE	Temocapril	2 mg/day	1	12	2.74	0.28	3.31	0.36
				Candesartan	8 mg/day	1	12	2.65	0.3	2.71	0.43
Akinboboye et al. (40)	HT&LVH	Clinical history and PET	PET	Lisinopril	10 mg/day	11	9	2.4	1	3.7	1.1
**NONRANDOMIZED**											
Galderisi et al. (41)	HT	Medical history	DE	Nebivolol	5 mg/day	3	20	2.07	0.16	2.2	0.243
Eshtehardi et al. (42)	SCAD	Invasive angiogram	IC Doppler	Atorvastatin	40–80 mg/day	6.5	20	2.32	0.44	2.53	0.89
Motz and Strauer (43)	HT with microvascular angina	Invasive angiogram	IC Doppler	Enalapril	10–20 mg/day	3	15	2.2	0.6	3.3	1.2
Caliskan et al. (44)	Slow coronary flow	Invasive angiogram	DE	Atorvastatin	20 mg/day	2	20	1.95	0.38	2.54	0.56
Galderisi et al. (45)	HT	Medical history	DE	Nebivolol	5 mg/day	1	14	1.89	0.31	2.12	0.33
Lethen et al. (46)	HT	Clinical history, ECG, and DE	PET	Irbesartan	600 mg/day	3	18	2.87	0.42	3.78	0.32
Tomás et al. (47)	HT	Clinical history, ECG, and DE	DE	Candesartan	16 mg/day	3	22	3.1	1	3.56	1
Sun et al. (48)	HT, HC	Medical history	DE	Rosuvastatin	10 mg/day	12	55	3.16	0.44	3.31	0.42
Jensen et al. (49)	HC	Invasive angiogram	IC Doppler	Simvastatin	40 mg/day	12	36	2.5	0.6	2.6	0.6
Baller et al. (50)	Angina	Invasive angiogram	PET	Simvastatin	20 mg/day	6.5	23	2.2	0.6	2.64	0.6
Schwartzkopff et al. (51)	HT	Invasive angiogram	DE	Perindopril	4–8 mg/day	12	14	2.1	0.6	3.5	1.9
Vogt and Strauer (52)	HT	Invasive angiogram	DE	Diltiazem	242 ± 35 mg/day	12	16	2.46	0.8	3.29	1.22
				Isradipine	5.3 ± 0.9 mg/day	12	15	2.33	0.55	3.3	0.87
Fujimoto et al. (53)	HC	Medical history	DE	Fluvastatin	20 mg/day	3	16	3	0.5	3.5	0.8
Stamatelopoulos et al. (54)	HT	Invasive angiogram	DE	Quinapril	20 mg/day	1	15	2.99	0.68	3.36	0.91
				Losartan	100 mg/day	1	15	2.86	0.54	3.44	0.65
Kjear et al. (55)	DM	Treadmill exercise test	PET	Losartan	100 mg/day	6.5	14	2.36	0.24	2.62	0.42
Kawata et al. (56)	DM	DE and a treadmill exercise test	DE	Temocapril	2 mg/day	1	20	2.78	0.36	3.35	0.46

*CMD, coronary microvascular dysfunction; DE, Doppler echocardiography; IC Doppler, intracoronary Doppler echocardiography; ECG, electrocardiography; PET, positron emission tomography; SPECT, single photon emission computed tomography; CMRI, coronary magnetic resonance imaging; SCAD, stable coronary artery disease; PCI, percutaneous coronary intervention; HT, hypertension; LVH, hypertrophy of left ventricular; HC, hypercholesterol; DM, diabetes mellitus*.

### Association of ACEIs and ARBs With CFR

**Figure 2:**
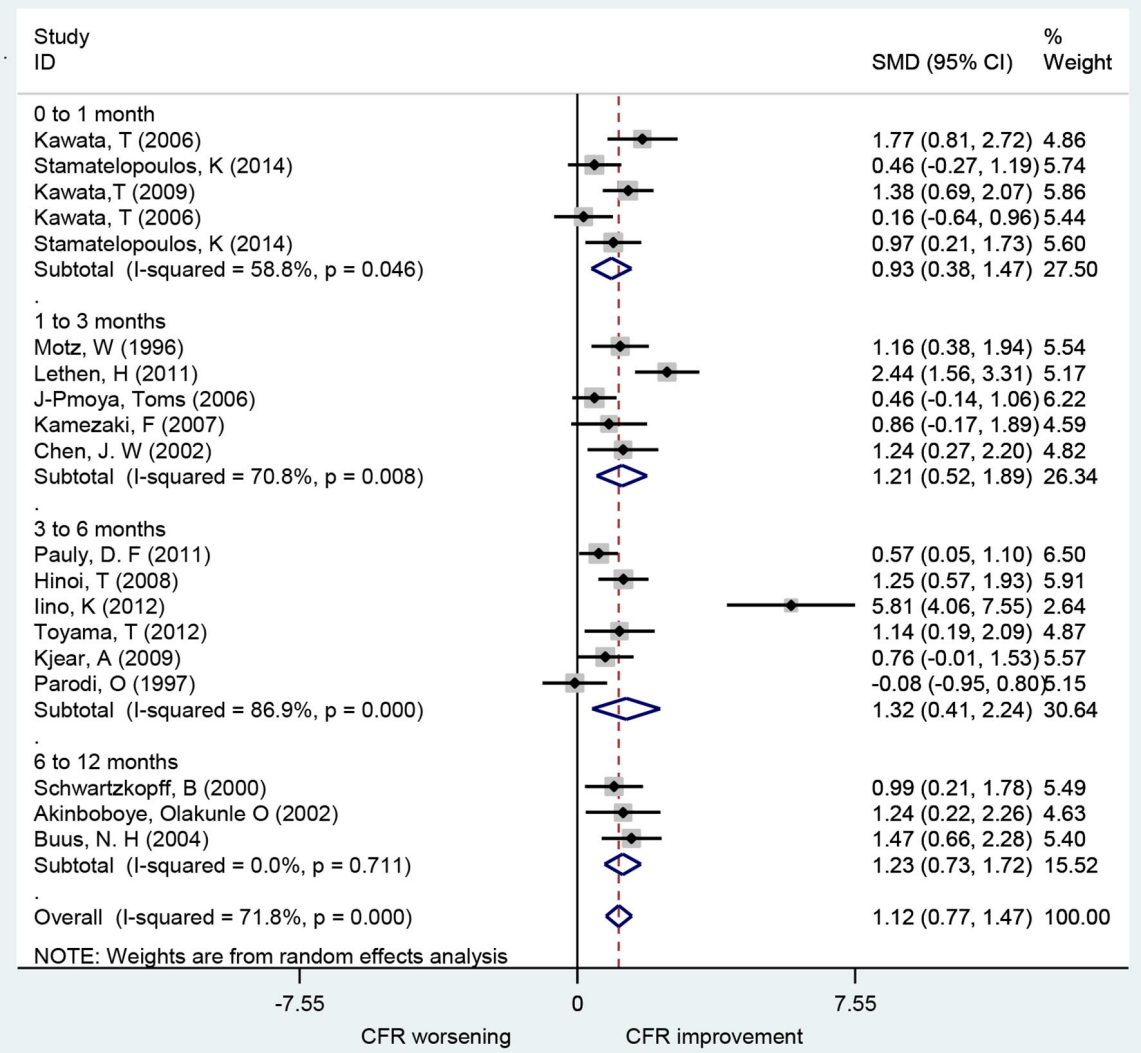
Forest plot of CFR for ACEI and ARB. CFR, coronary flow reserve; ACEI, aldosterone receptor antagonist; ARB, aldosterone receptor antagonist.

A total of 19 studies investigated the effect of ACEIs and ARBs on CFR improvement, including 10 for ACEIs and 9 for ARBs. The CFRs in patients receiving either ACEIs or ARBs improved significantly compared to baseline with a pooled estimate SMD of 1.12 [95% confidence interval (CI): 0.77–1.47, *I*^2^ = 71.8%] (Figure 2). Changes in CFR across the follow-up period subgroups were similar: 0–1 month (SMD: 0.93; 95% CI: 0.38–1.47, *I*^2^ = 58.8%); 1–3 months (SMD: 1.21; 95% CI: 0.52–1.89, *I*^2^ = 70.8%); 3–6 months (SMD: 1.32; 95% CI: 0.41–2.24, *I*^2^ = 86.9%); 6–12 months (SMD: 1.23; 95% CI: 0.73–1.72, *I*^2^ = 0.0%). For patients with angina symptoms, ACEIs and ARBs can improve the CFR (SMD: 1.95; 95% CI: 0.53–3.38, *I*^2^ = 90.6%) (**Figure 7**). These findings indicate that treatment with ACEIs or ARBs may improve CFR for patients without obstructive CAD.

### Association of Beta-Blocker With CFR

**Figure 3:**
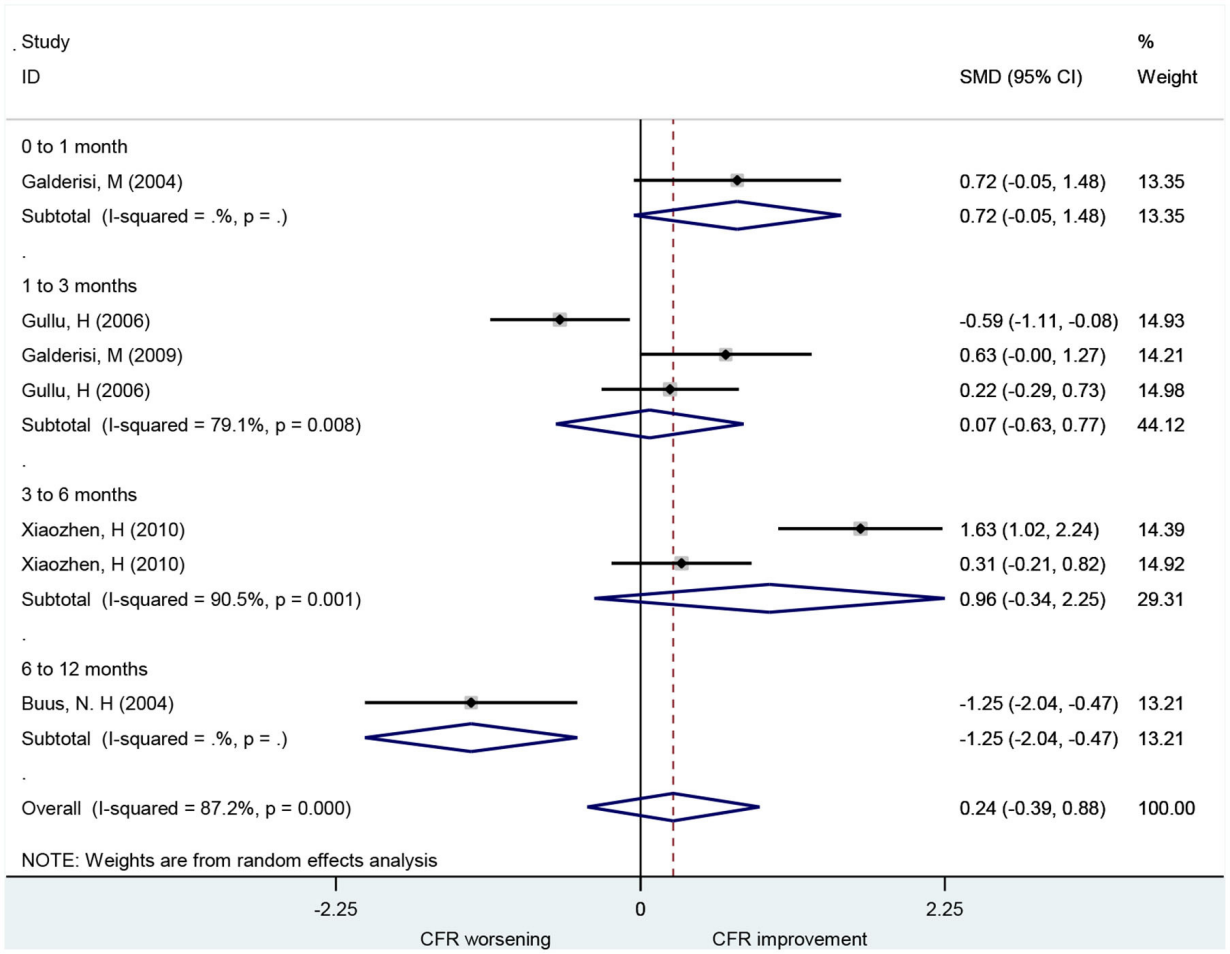
Forest plot of CFR for beta-blockers. CFR, coronary flow reserve.

A total of seven studies that included 166 patients investigated the effects of beta-blocker on CFR. These found no difference in CFR at follow-up compared to baseline (SMD: 0.24; 95% CI: −0.39–0.88, *I*^2^ = 87.2%) (Figure 3). There was no statistical difference in CFR change across subgroups for follow-up periods up to 6 months: 0–1 month (SMD: 0.72; 95% CI: −0.05–1.48, *I*^2^ =. %); 1–3 months (SMD: 0.07; 95% CI: −0.63–0.77, *I*^2^ = 79.1%); 3–6 months (SMD: 0.96; 95% CI: −0.34–2.25, *I*^2^ = 90.5%). Change in CFR was significant for the 6–12 months follow-up subgroup; however, only one study reported data on this subgroup (SMD: −1.25; 95% CI: −2.04 to −0.47, *I*^2^ =. %). For patients with myocardial ischemic symptoms, beta-blockers had no significant effect on CFR (SMD: 0.96; 95% CI: −0.34–2.25, *I*^2^ = 90.5%) (**Figure 7**).

### Association of CCB With CFR

**Figure 4:**
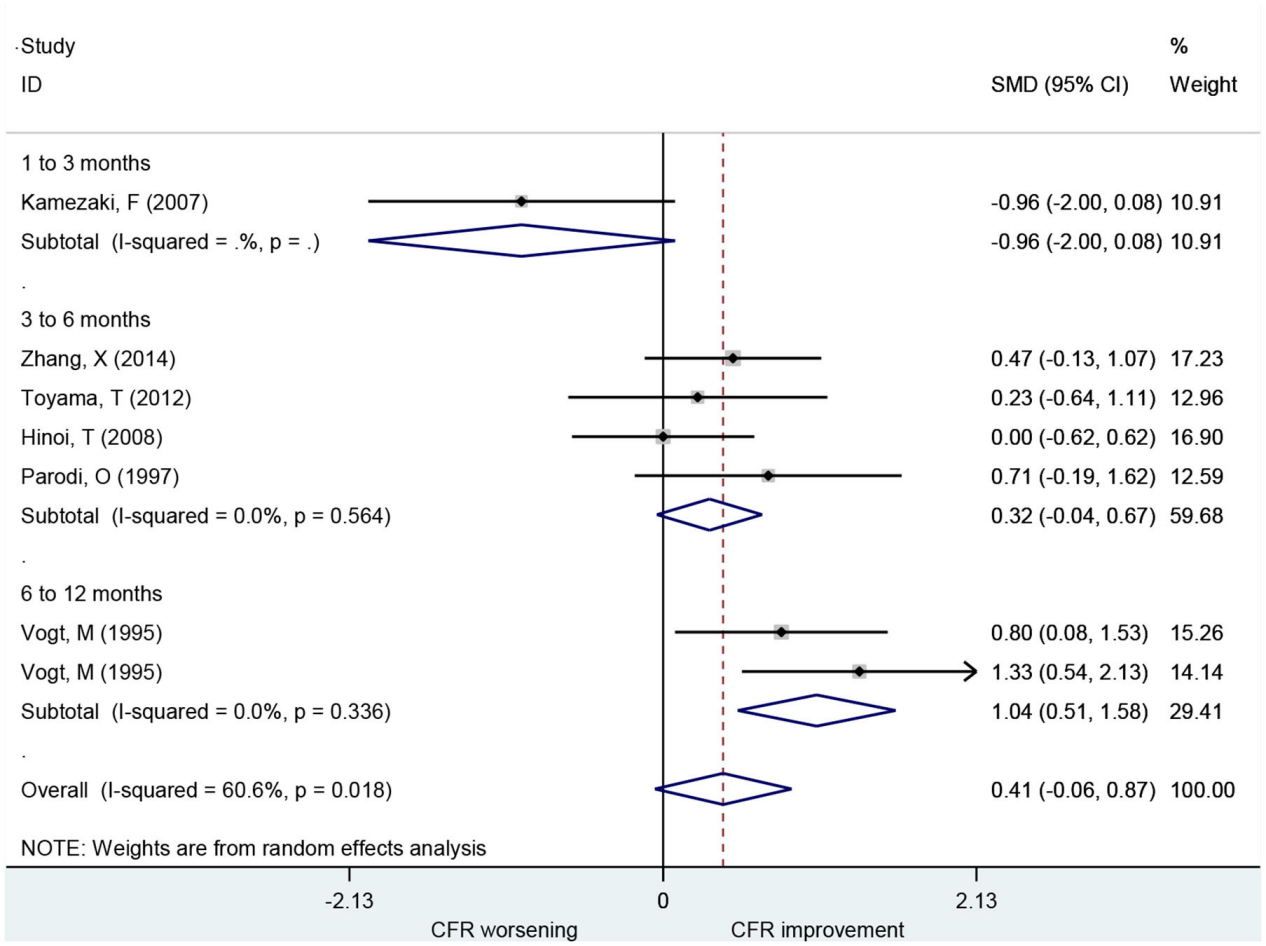
Forest plot of CFR for CCBs. CFR, coronary flow reserve; CCBs, calcium channel blockers.

A total of seven studies that included 101 patients investigated the use of CCBs for CFR improvement. They showed no difference in CFR at follow-up compared to baseline (SMD: 0.41; 95% CI: −0.06–0.87, *I*^2^ = 60.6%) (Figure 4). Changes in CFR were not significantly different across follow-up subgroups for periods up to 6 months: 1–3 months (SMD: −0.96; 95% CI: −2.00–0.08, *I*^2^ =. %); 3–6 months (SMD: 0.32; 95% CI: −0.04–0.67, *I*^2^ = 0%). However, CCBs were associated with CFR improvement in the 6–12 months follow-up subgroup (SMD: 1.04; 95% CI: 0.51–1.58, *I*^2^ = 0%).

### Association of Ranolazine With CFR

**Figure 5:**
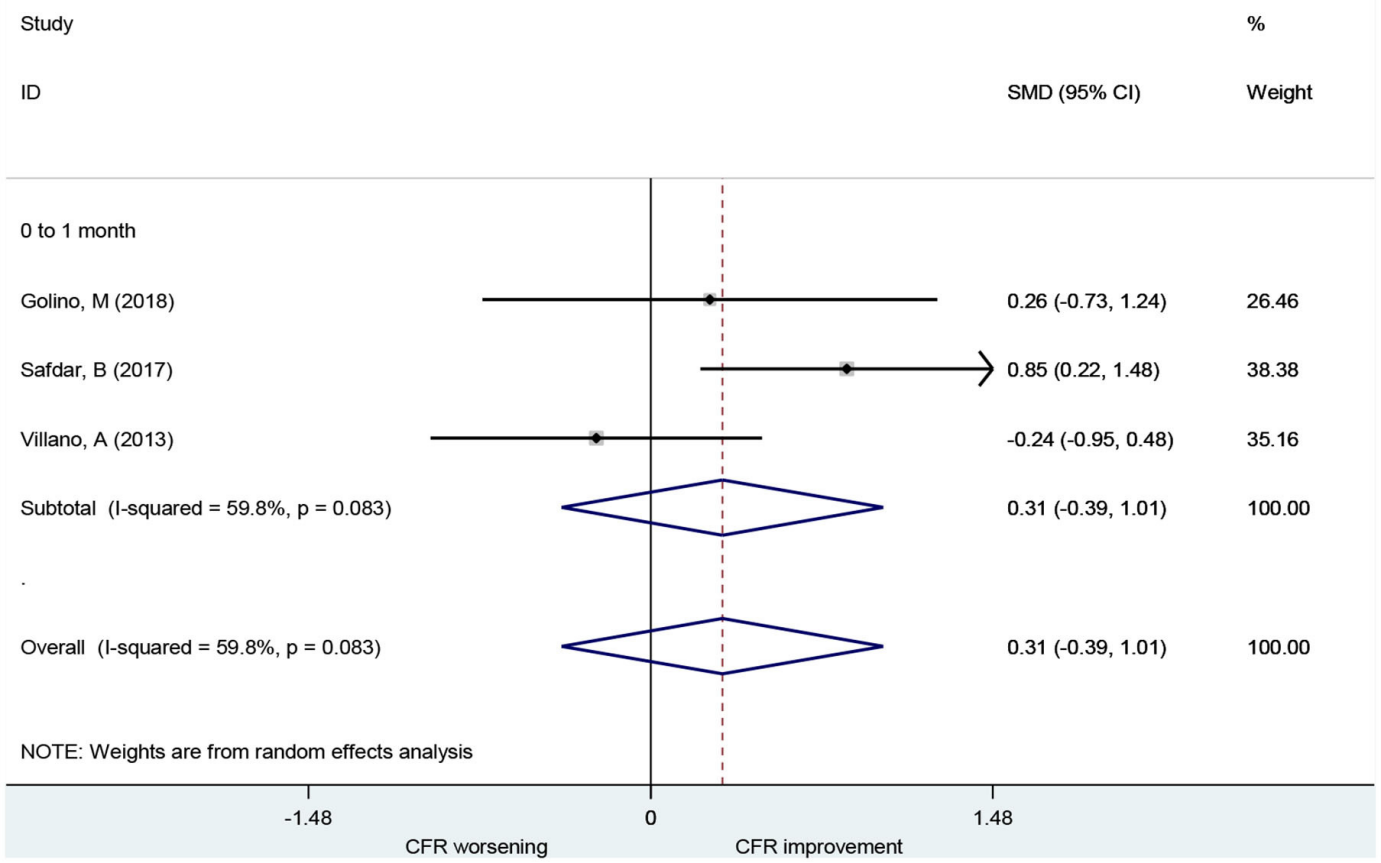
Forest plot of CFR for ranolazine. CFR, coronary flow reserve.

A total of three studies including 44 patients investigated the use of ranolazine for CFR improvement. All patients included had symptoms of myocardial ischemia. CFR was not improved significantly after patients received ranolazine for 1 month (SMD, 0.31; 95% CI: −0.39–1.01, *I*^2^ = 59.8%) (Figure 5).

### Association of Statins With CFR

**Figure 6:**
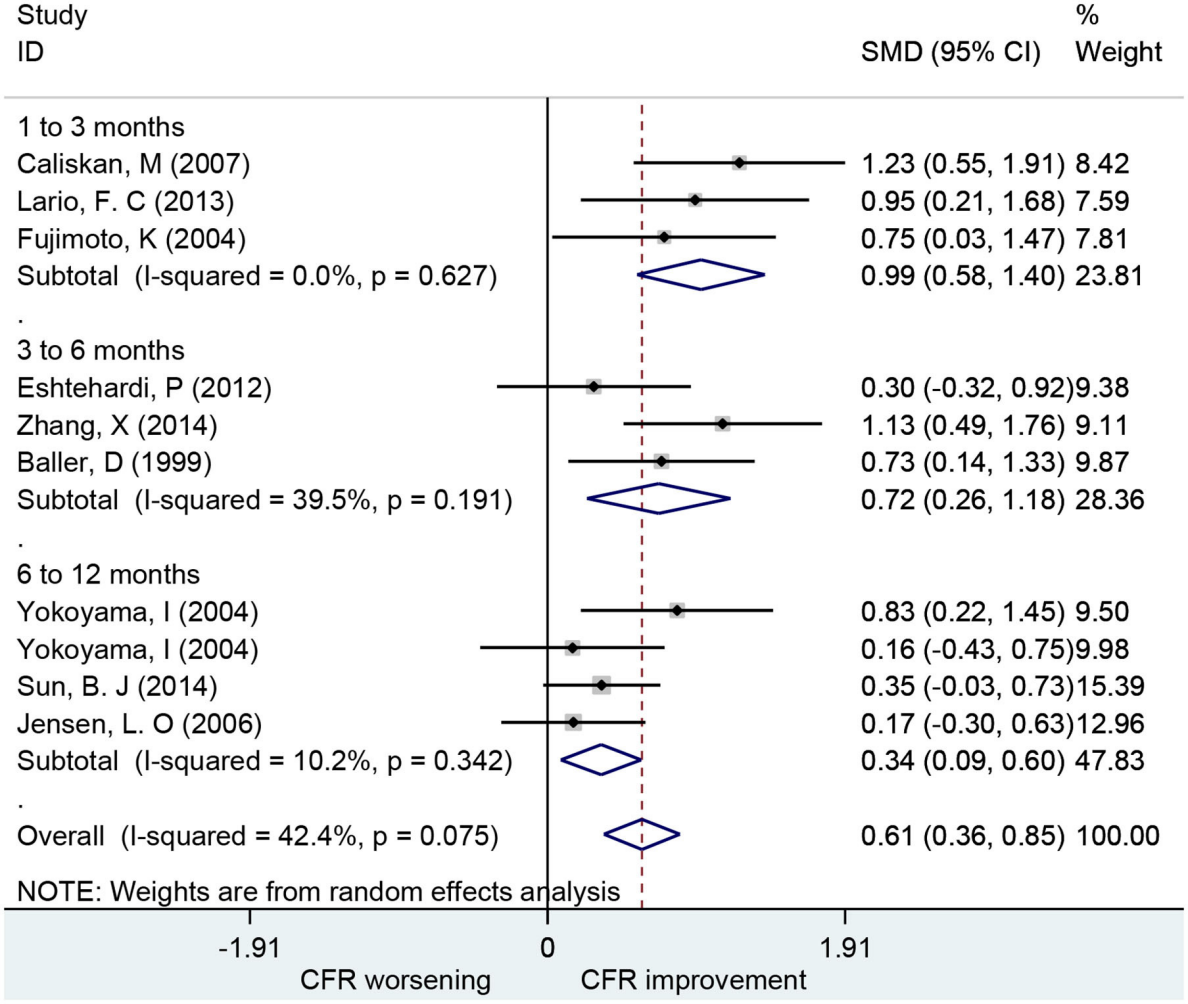
Forest plot of CFR for statin. CFR, coronary flow reserve.

**Figure 7:**
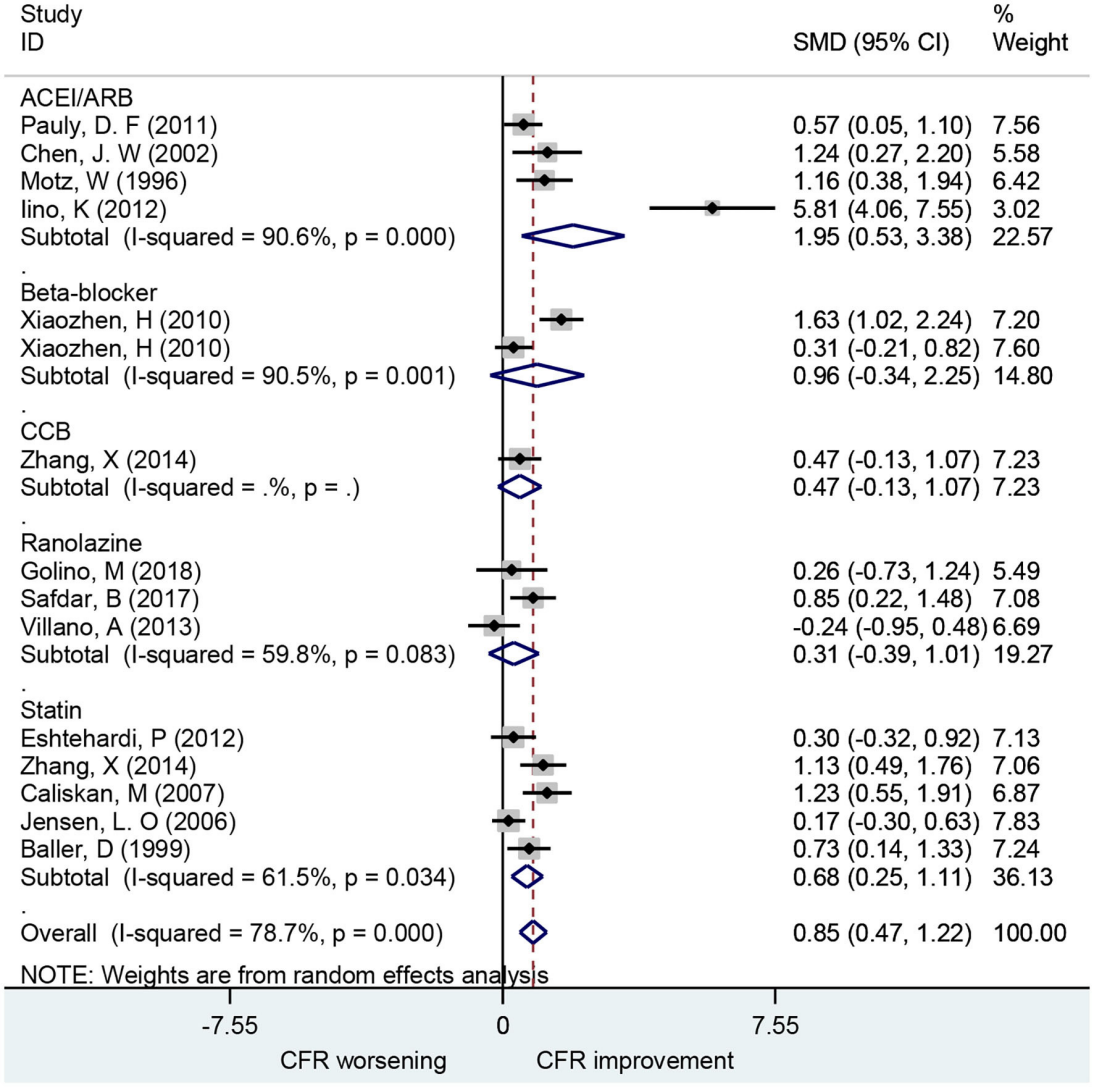
Forest plot of CFR of different drugs in patients with myocardial ischemia symptoms. CFR, coronary flow reserve; ACEI, aldosterone receptor antagonist; ARB, aldosterone receptor antagonist; CCB, calcium channel blocker.

Ten studies including 252 patients investigated the use of statins for CFR improvement. Patients receiving statins showed significant improvement in CFR compared to baseline with a pooled estimate SMD of 0.61 (95% CI: 0.36–0.85, *I*^2^ = 42.4%) (Figure 6). Subgroup analyses by follow-up time showed that changes in CFR were similar: 1–3 months (SMD: 0.99; 95% CI: 0.58–1.40, *I*^2^ = 0%); 3–6 months (SMD: 0.72; 95% CI: 0.26–1.18, *I*^2^ = 39.5%); 6–12 months (SMD: 0.34; 95% CI: 0.09–0.60, *I*^2^ = 10.2%). Five of these studies showed that statins can improve CFR in patients with symptoms of myocardial ischemia (SMD: 0.68; 95% CI: 0.25–1.11, *I*^2^ = 78.7%) (Figure 7). This indicates that statins may improve CFR for patients without coronary stenosis, regardless of follow-up period duration.

### Quality of Studies, Clinical Heterogeneity, and Publication Bias

The quality assessment of included studies is shown in Supplemental Table 3. The quality of the 18 studies that mentioned randomized grouping was primarily assessed. Three studies were considered low risk with respect to randomization (9, 23, 27) because they used computer-designated procedure, and the remaining studies were classified as unclear risk as they did not elaborate on the randomized methods applied. Except for the study of Pauly et al. no other randomized controlled studies reported location concealment in detail and were therefore considered unclear risk. As for the performance bias, 12 randomized controlled studies were considered low risk as they were blinded, and the remaining six studies reported no blinding or unclear blinding methods. For eight randomized controlled studies, no treatment or distribution schemes were known by the outcome observers, and a third party performed outcome analyses. Therefore, detection bias was considered low risk. Attrition bias was considered low risk because the final proportion of those who completed the study in both groups was almost the same as that in the beginning despite participant dropout. Neither reporting bias nor other biases were found in any included randomized controlled study. In addition, we restricted analyses to 17 studies with high methodological quality, as indicated by a Newcastle–Ottawa Scale (NOS) score of 60% or more.

In terms of CFR, statistical heterogeneity as measured by *I*^2^ ranged from 42.04 to 87.2%, indicating no to moderate heterogeneity. Some heterogeneity can be explained by differences in follow-up duration, drug dosage, study type, imaging methods used, and patient disease. Considering the potentially high degree of heterogeneity between studies, a subgroup analysis was performed based on follow-up duration. In the sensitivity analysis, the conclusion from recalculating pooled estimates were consistent with the primary analysis, when each study was excluded individually. This was reflected by the 95% CIs of the separate studies that overlap well (Supplementary Figures 1–5).

We found evidence of publication bias based on the funnel plots (Supplementary Figure 6) or the Egger test (t = 3.04; P = 0.004).

## Discussion

We reviewed 46 studies that assessed CFR improvement in 845 patients without obstructive CAD treated with oral drugs. We found that ACEIs, ARBs, and statins were associated with significant improvement in CFR compared to baseline among patients who were followed up between 0 and 12 months. Moreover, CCBs and β-blockers were also associated with improved CFR when follow-up was extended to 6–12 months. Treatment with ranolazine for 1 month had no significant effect on CFR improvement compared to baseline. Compared with the previous studies, the present study focused on the improvement of microvascular function by oral medication during different follow-up periods.

The 2020 ESC position paper on CMD and 2019 ESC guidelines on chronic coronary syndromes recommend that ACEI/ARB, statins, and beta-blockers can be used for secondary prevention treatment of CMD (17). Several reviews suggest exercise, control of risk factors, and drugs such as ACEIs/ARBs and statins as effective first-line treatments and CCBs, nicorandil, and ranolazine as second-line treatments (8, 14, 18, 19). However, these recommendations are controversial regarding the therapeutic effects of beta-blockers, CCB, and ranolazine and did not give a definite treatment time. Our research affirmed that long-term CCB improved CFR, but beta-blockers and ranolazine did not. We also conducted subgroup analysis of follow-up time, which allowed better guidance of clinical medication time.

Previous studies have found that ACEIs and ARBs can improve microcirculatory function, which corresponds with the findings of this study (9, 54, 57, 58). Studies evaluating the effects of ACEIs and ARBs on Seattle Angina score and E/e' indicate that the CFR improvement is associated with reduced angina and left ventricular filling pressures (9, 30, 54). This mechanism by which ACEIs and ARBs improve CFR may be related to their anti-inflammatory and antioxidant characters as well as their effects of coronary endothelial dysfunction improvement and vasodilatation (29, 54, 59). Thus, ACEI improvements in microcirculatory function and left ventricular diastolic function are not merely dependent on its antihypertensive and antiventricular remodeling effects (46, 54).

We found that early treatment with statins was associated with improved microcirculatory function, similar with the findings of previous studies (38, 42, 48). The mechanisms by which statins protect microcirculatroy function include their anti-inflammatory effect, endothelium protective effect, and antiremodeling effect on left ventricular (60–64). The benefits of statin therapy may also be related to elevated levels of nitric oxide and reduced expression of endothelin-1 (28, 61). These findings suggest that short-term statin therapy is recommended to improve coronary microvascular function.

CCBs showed improved CFR among patients followed up for 0–6 months, but the drug was found to be effective with prolonged follow-up time (34, 41, 65). Therefore, the negative findings from previous studies may be due to insufficient treatment duration (30, 31, 33, 66). CCBs may improve microcirculation through vasodilation.

This study found that β-blockers did not improve CFR. However, many studies have shown that β-blockers have microcirculation protection, which was attributable to its antioxidant and endothelial protection properties (41, 45, 67, 68). We found that ranolazine did not improve CFR, and other studies have yielded similar findings (23, 26, 69–71). However, some studies indicate that ranolazine may improve the coronary microvascular function by improving angina symptoms (18, 72). This inconsistency in study findings may be due to insufficient dosage and treatment time (73). Ranolazine exerts its anti-ischemic effect by affecting the activity of pyruvate dehydrogenase to improve myocardial energy metabolism, by inhibiting the late Na^+^ current in myocardial cells, and reducing Ca^2+^ influx, thereby improving diastolic function and myocardial perfusion. However, this is an effect that can only be achieved at high concentrations (23, 26). Additional large-scale follow-up studies are needed to fully clarify this.

Moreover, some studies indicate that nicorandil may improve coronary microvascular function by regulating plasma levels of nitric oxide and endothelin-1 (74–77). However, most of these studies involved intracoronary injections or intravenous drugs and were therefore excluded from evaluation here. Ivabradine and diuretics are also reported to be associated with improved of microcirculation function (12, 18, 78). However, a meta-analysis could not be performed due to the small number of available studies.

Several studies have investigated the effects of medication on CMD without obstructive CAD and concluded that optimal treatment for microvascular angina should focus on relieving symptoms and improving vascular function (18). Exercise and weight loss, in addition to statins, L-arginine, ACEIs, and beta-blockers, can also improve vascular function via restoring endothelial dysfunction and impaired CFR (18). Effective treatment for microvascular angina requires aggressive control of risk factors, and one of the most effective methods is exercise. Further studies should be carried out to determine whether specific treatments are associated with prolonged survival and symptom alleviation (18).

Comparisons of IMR, fractional flow reserve (FFR), coronary microvascular resistance (CMR), myocardial blood flow (MBF), myocardial perfusion reserve index (MPRI), and CFR indicate that CFR is more available for coronary microvascular function evaluation (14, 19, 68). Methods to assess CFR include coronary angiogram, PET, CMR, and transthoracic Doppler echocardiography (TTDE) (79). During coronary angiogram, CFR measurement is made using a Doppler guidewire following administration of intracoronary adenosine (9). During TTDE, PET, and CMR, coronary flow velocity at rest and maximal hyperemia induced by administration of intravenous adenosine (140 lg/kg/min) are recorded to measure CFR (79). Studies have established CFR ranges to assess coronary physiology in patients with an increased risk of coronary heart disease or symptoms of chest pain but with normal coronary angiography (17, 23, 69). Variations in study findings may arise from differences in the indicators selected for evaluation. To avoid this, we chose CFR as the primary and only indicator to evaluate coronary microvascular function improvement.

### Limitations

**Figure 8:**
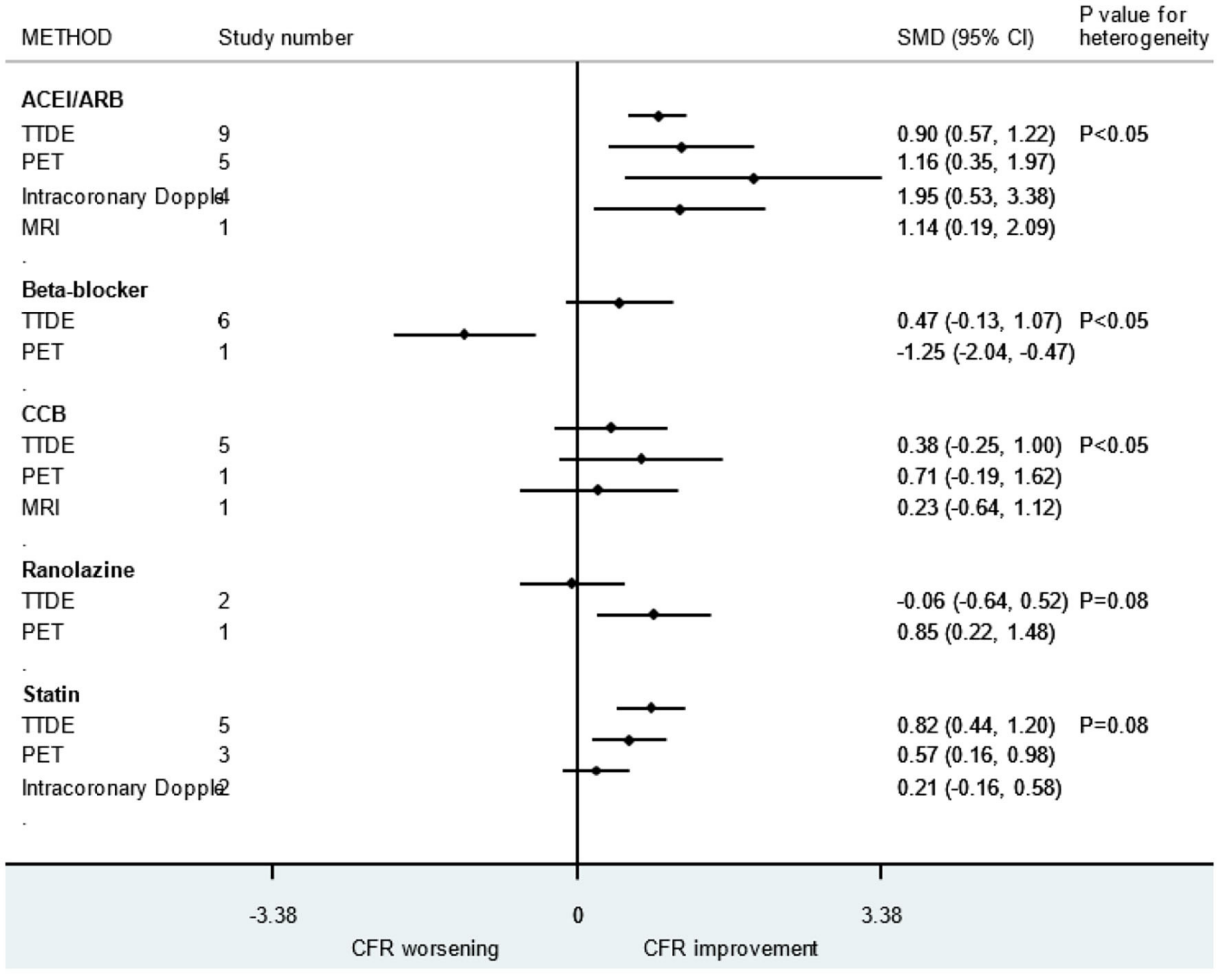
Subgroup analysis of different CFR detection methods. ACEI, aldosterone receptor antagonist; ARB, aldosterone receptor antagonist; CCB, calcium channel blocker; CFR, coronary flow reserve.

First, due to the fact that we focused on the improvement of microcirculation function by drugs among patients without obvious coronary stenosis, not all patients in this study had CMD (symptoms of chest pain and abnormal CFR). The CFR varied greatly due to the diagnosis and condition of the patients. Second, there was no uniform detection method for measuring CFR and the sensitivity, effectiveness, and accuracy of different detection methods varied across studies. In addition, we realized that despite the same detection methods, the use of different test drugs or different administration methods during measurement can also cause differences. We conducted subgroup analysis based on different detection methods and found that there are indeed differences between the different detection methods (Figure 8). Finally, improvement of symptoms is also an important indicator for evaluating microcirculation disorders. However, due to the fact that only limited numbers of retrieved studies used symptoms as an outcome, we only choose CFR in this meta-analysis.

## Conclusion

Statin, ACEI, and ARB therapy could improve CFR in patients without obstructive CAD from 0 to 12 months. CCBs and beta-blockers were associated with improved CFR in patients followed for 6–12 months. One month of treatment with ranolazine was not associated with improve CFR.

## Data Availability Statement

All datasets generated for this study are included in the article/Supplementary Material.

## Author Contributions

XS and YH helped to conceive the topic and revise the manuscript. JY and JT contributed to the study selection, data extraction, and manuscript draft. XY and HX contributed to the data analysis. All authors contributed to the article and approved the submitted version.

## Conflict of Interest

The authors declare that the research was conducted in the absence of any commercial or financial relationships that could be construed as a potential conflict of interest.
