# Supplementary material for: Effects of Oral Drugs on Coronary Microvascular Function in Patients Without Significant Stenosis of Epicardial Coronary Arteries: A Systematic Review and Meta-Analysis of Coronary Flow Reserve
Source: Front Cardiovasc Med. 2020 Oct 30;7:580419. doi: 10.3389/fcvm.2020.580419 (PMC7661556; doi:10.3389/fcvm.2020.580419)
Supplement: Supplementary file 1 [file Data_Sheet_1.docx]

**Appendix**

**Supplementary data**

Supplementary Figure 1. Sensitivity analysis of ACEI and ARB.

ACEI, aldosterone receptor antagonist; ARB, aldosterone receptor antagonist.

Supplementary Figure 2. Sensitivity analysis of Beta-blocker.

Supplementary Figure 3. Sensitivity analysis of CCB.

CCB, calcium channel blocker.

Supplementary Figure 4. Sensitivity analysis of Ranolazine.

Supplementary Figure 5. Sensitivity analysis of Statin.

Supplementary Figure 6. Publication bias.

Supplementary Table 1. Search terms used to identify articles for review

| **Database** | **Search terms** |
| --- | --- |
| PubMed | Heading [MeSH terms], search filter [title/abstract] and other terms as follows:  ((CFR OR coronary flow reserve OR coronary flow OR myocardial perfusion OR myocardial blood flow OR myocardial perfusion reserve OR coronary flow velocity reserve[MeSH Terms]) AND (microvascular dysfunction OR microvessels dysfunction OR microcirculation[MeSH Terms])) AND (treatment[Title/Abstract] OR management[Title/Abstract] OR therapy[Title/Abstract] OR oral drug*[Title/Abstract] OR Pharmacotherapy[Title/Abstract] OR nicorandil[Title/Abstract] OR Fasudil[Title/Abstract] OR mibefradil[Title/Abstract] OR ATP-sensitive potassium channel openers[Title/Abstract] OR randolazine[Title/Abstract] OR ivabradine[Title/Abstract] OR ACE-inhibitors[Title/Abstract] OR Angiotensin-converting enzyme inhibitor[Title/Abstract] OR ACEI[Title/Abstract] OR Enalapril[Title/Abstract] OR Perindopril[Title/Abstract] OR Ramipril[Title/Abstract] OR pril[Title/Abstract] OR sartan[Title/Abstract] OR candesartan[Title/Abstract] OR Telmisartan[Title/Abstract] OR Valsartan[Title/Abstract] OR Olmesartan[Title/Abstract] OR Angiotensin receptor blocker[Title/Abstract] OR ARB[Title/Abstract] OR trimetazidine[Title/Abstract] OR statin[Title/Abstract] OR beta Blocker[Title/Abstract] OR olol[Title/Abstract] OR bisoprolol[Title/Abstract] OR Carvedilol[Title/Abstract] OR Metoprolol[Title/Abstract] OR Nebivolol[Title/Abstract] OR Atenolol[Title/Abstract] OR calcium-channel blocker[Title/Abstract] OR dipin[Title/Abstract] OR Amlodipin[Title/Abstract] OR Nifedipine[Title/Abstract] OR Verapamil[Title/Abstract] OR Nifedipine[Title/Abstract] OR Diltiazem[Title/Abstract] OR Nitrate[Title/Abstract]) |
| EMBASE | (‘CFR’/exp OR ‘coronary flow reserve’/exp OR ‘coronary flow’/exp OR ‘myocardial perfusion’ OR ‘myocardial blood flow’ OR ‘myocardial perfusion reserve’ OR ‘coronary flow velocity reserve’) AND (‘microvascular dysfunction’/exp OR ‘microvessels dysfunction’/exp OR ‘microcirculation’/exp) |
| Cochrane Library | “coronary flow reserve” AND “microvascular dysfunction” |
|  |  |

Supplementary Table 2. Patient characteristics

| **Author** | **Drugs** | **N** | **Age** | **Male** | **SBP** | **DBP** | **HR** | **BMI** | **Smoking** | **HT** | **DM** | **DL** |
| --- | --- | --- | --- | --- | --- | --- | --- | --- | --- | --- | --- | --- |
| **Randomized** |  |  |  |  |  |  |  |  |  |  |  |  |
| Golino, M, et al. 2018 | Ranolazine/ISMN | 15 | 67.3±5.4 | 14 | 126±10 | 73±7 | 68±9 | 26.3±2.9 | NA | 12 | 7 | 15 |
| Safdar, B, et al. 2017 | Ranolazine | 21 | 50±7 | 8 | 130±18 | NA | 73±10 | 43±10 | 13 | 15 | 5 | 5 |
| Villano, A, et al. 2013 | Ranolazine | 15 | 57±11 | 3 | 153±17 | NA | 116±19 | 27±4 | 2 | 13 | NA | 8 |
| Zhang, X, et al. 2014 | Diltiazem | 22 | 54±8 | 10 | 128±19 | 74±10 | NA | NA | NA | NA | NA | NA |
| Zhang, X, et al. 2014 | Fluvastatin | 22 | 54±7 | 10 | 128±20 | 72±11 | NA | NA | NA | NA | NA | NA |
| Pauly, D. F, et al. 2011 | Quinapril | 29 | 56±8 | 0 | 126±15 | 73±8 | 72±12 | 29.4±5.7 | 34 | 34 | 17 | 61 |
| Iino, K, et al. 2012 | Candesartan | 14 | 69.1±5.8 | 11 | 134±14 | 73.2±8.7 | NA | NA | 7 | NA | NA | NA |
| Chen, J. W, et al. 2002 | Enalapril | 10 | 66.3±3.5 | 8 | 136±20 | 79±12 | 72±18 | NA | NA | NA | NA | NA |
| Toyama, T, et al. 2012 | Olmesartan | 10 | 52.2±9.2 | 6 | 178±22.2 | 104.1±16.8 | 73.7±5.9 | 24.1±4.1 | 8 | NA | NA | NA |
| Toyama, T, et al. 2012 | Amlodipin | 10 | 58.9±13.3 | 7 | 174.3±20.9 | 105.3±15.8 | 73.0±12 | 25.5±3.8 | 5 | NA | NA | NA |
| Kamezaki, F, et al. 2007 | Valsartan | 8 | 64.4±8.5 | 6 | 155.5±18.7 | 94.3±14.9 | 71±7 | 24.2±3.1 | 2 | NA | 1 | 7 |
| Kamezaki,F, et al. 2007 | Nifedipine | 8 | 61.6±11.3 | 4 | 151.3±9.9 | 87.5±8.9 | 69±6 | 26.5±4.1 | 1 | NA | 2 | 4 |
| Parodi, O, et al.1997 | Enalapril | 10 | 52±8 | 6 | 164±16 | 103±6 | 63±10 | NA | NA | NA | NA | NA |
| Parodi, O, et al. 1997 | Verapamil | 10 | 54±6 | 7 | 167±11 | 105±11 | 65±8 | NA | NA | NA | NA | NA |
| Hinoi, T, et al. 2008 | Telmisartan | 20 | 62±7 | 8 | NA | NA | NA | 24±1.5 | 2 | 0 | 0 | 20 |
| Hinoi, T, et al. 2008 | Nifedipine | 20 | 63±8 | 9 | NA | NA | NA | 23±1.2 | 2 | 0 | 0 | 20 |
| Xiaozhen, H, et al. 2010 | Carvedilol | 28 | 60.2±11.6 | 18 | 156±7 | 158±4 | 89±4 | NA | NA | 28 | NA | NA |
| Xiaozhen, H, et al. 2010 | Metoprolol | 29 | 62.1±13.8 | 17 | 90±2 | 89±3 | 90±2 | NA | NA | 29 | NA | NA |
| Gullu, H, et al. 2006 | Nebivolol | 30 | 41.4±6.0 | 12 | 146±7 | 90±4 | 73.13±11.67 | NA | NA | NA | NA | NA |
| Gullu, H, et al. 2006 | Atenolo | 30 | 41.4±6.0 | 12 | 149±8 | 89±4 | 74.08±11.45 | NA | NA | NA | NA | NA |
| Buus, N. H, et al. 2004 | Perindopril | 15 | 49±2 | 10 | 153±2 | 100±2 | 81±2 | 27.1±0.5 | NA | NA | NA | NA |
| Buus, N. H, et al. 2004 | Atenolol | 15 | 51±2 | 12 | 152±3 | 101±2 | 75±3 | 26.6±0.6 | NA | NA | NA | NA |
| Yokoyama, I, et al. 2004 | Simvastatin | 22 | 51.8±9.1 | 12 | 132.9±19.5 | 82.0±13.9 | 64.9±10.5 | NA | NA | NA | NA | NA |
| Yokoyama, I, et al. 2004 | Pravastatin | 22 | 55.9±9.7 | 13 | 136.6±18.5 | 76.7±13.0 | 65.2±11.8 | NA | NA | NA | NA | NA |
| Lario, F. C, et al. 2013 | Atorvastatin | 16 | 39±14 | 7 | NA | NA | NA | 24±3 | 3 | 1 | 0 | 16 |
| Kawata, T, et al. 2006 | Temocapril | 12 | 65±12 | 9 | 127.1±23.3 | 64.9±10.1 | 69.6±12 | 22.3±3 | 0 | 4 | 12 | NA |
| Kawata, T, et al. 2006 | Candesartan | 12 | 67±9 | 8 | 131.2±18.2 | 66.2±10.7 | 73.0±16.6 | 23.2±2.7 | 0 | 4 | 12 | NA |
| Akinboboye, O, et al. 2002 | Lisinopril | 9 | 55±2 | 5 | 160±16 | 93±16 | 83±8 | 32±5 | NA | 6 | NA | NA |
| **Nonrandomized** |  |  |  |  |  |  |  |  |  |  |  |  |
| Galderisi, M, et al. 2009 | Nebivolol | 20 | 49.7 | 16 | 148.3±7.9 | 101.4±4.6 | 77.6±5.9 | 28.3±2.4 | NA | NA | NA | NA |
| Eshtehardi, P, et al. 2012 | Atorvastatin | 20 | 54(46 to 68) | 13 | 129(114 to 145) | 72(68 to 83) | NA | 30(27 to 36) | 5 | 14 | 7 | 20 |
| Motz, W, et al. 1996 | Enalapril | 15 | 58±6 | 10 | 178±14 | 102±11 | 69±9 | NA | NA | 15 | NA | NA |
| Caliskan, M, et al. 2007 | Atorvastatin | 20 | 54.7±12.1 | 28 | 123.3±8.2 | 77±4.7 | 66.3±8.1 | 28±3 | NA | NA | NA | NA |
| Galderisi, M, et al. 2004 | Nebivolol | 14 | 47.4 | 10 | 148.2±9.1 | 101.4±4.6 | 77.0±6.9 | 28.5±2.7 | NA | 14 | 0 | NA |
| Lethen, H, et al. 2011 | Irbesartan | 18 | 54±9 | 18 | 150±18 | 88±11 | NA | NA | NA | 18 | 3 | 7 |
| J-Pmoya, Toms, et al. 2006 | Candesartan | 22 | 63.86±10.3 | 9 | 143±17.9 | 86.36±9.4 | NA | NA | NA | 22 | NA | 22 |
| Sun, B. J, et al. 2014 | Rosuvastatin | 55 | 61±9 | 40 | 115±13 | 68±9 | 68±12 | NA | NA | NA | NA | NA |
| Jensen, L. O, et al. 2006 | Simvastatin | 36 | 57.8±9.1 | 36 | NA | NA | NA | 27.5±3.6 | 15 | 9 | 0 | 36 |
| Baller, D, et al. 1999 | Simvastatin | 23 | 56±7.6 | 18 | NA | NA | NA | 26.6±2.3 | 9 | 8 | 0 | 23 |

CFR, coronary flow reserve; SBP, systolic blood pressure; DBP, diastolic blood pressure; BMI, body mass index; HT, hypertension; DM, diabetes mellitus; DL, dyslipidemia.

Supplementary Table 3. Study Quality Assessment

| ***Randomized Controlled Trials*** | | | | | | | | |
| --- | --- | --- | --- | --- | --- | --- | --- | --- |
| **Author** | **Random sequence generation (selection bias)** | **Allocation concealment (selection bias)** | **Blinding of participants and personnel (performance bias)** | **Blinding of outcome data (detection bias)** | **Incomplete outcome data (attrition bias)** | **Selective reporting (reporting bias)** | **Other bias** |  |
| Golino, M,et al.2018 | UNCLEAR | UNCLEAR | LOW | UNCLEAR | LOW | LOW | LOW |  |
| Safdar, B,et al.2017 | LOW | UNCLEAR | LOW | LOW | LOW | LOW | LOW |  |
| Villano, A,et al.2013 | LOW | UNCLEAR | LOW | LOW | LOW | LOW | LOW |  |
| Zhang, X,et al.2014 | UNCLEAR | UNCLEAR | HIGH | HIGH | LOW | LOW | LOW |  |
| Pauly, D. F,et al.2011 | LOW | LOW | LOW | LOW | LOW | LOW | LOW |  |
| Iino, K,et al.2012 | UNCLEAR | UNCLEAR | HIGH | HIGH | LOW | LOW | LOW |  |
| Chen, J. W,et al.2002 | UNCLEAR | UNCLEAR | LOW | UNCLEAR | LOW | LOW | LOW |  |
| Toyama, T,et al.2012 | UNCLEAR | UNCLEAR | HIGH | HIGH | LOW | LOW | LOW |  |
| Kamezaki, F,et al.2007 | UNCLEAR | UNCLEAR | LOW | LOW | LOW | LOW | LOW |  |
| Parodi, O,et al.1997 | UNCLEAR | UNCLEAR | LOW | LOW | LOW | LOW | LOW |  |
| Hinoi, T,et al.2008 | UNCLEAR | UNCLEAR | LOW | LOW | LOW | LOW | LOW |  |
| Xiaozhen, H,et al.2010 | UNCLEAR | UNCLEAR | LOW | LOW | LOW | LOW | LOW |  |
| Gullu, H,et al.2006 | UNCLEAR | UNCLEAR | LOW | UNCLEAR | LOW | LOW | LOW |  |
| Buus, N. H,et al.2004 | UNCLEAR | UNCLEAR | LOW | UNCLEAR | LOW | LOW | LOW |  |
| Yokoyama, I,et al.2004 | UNCLEAR | UNCLEAR | HIGH | HIGH | LOW | LOW | LOW |  |
| Lario, F. C,et al.2013 | UNCLEAR | UNCLEAR | HIGH | HIGH | LOW | LOW | LOW |  |
| Kawata, T,et al.2006 | UNCLEAR | UNCLEAR | LOW | LOW | LOW | LOW | LOW |  |
| Akinboboye, Olakunle O,et al.2002 | UNCLEAR | UNCLEAR | HIGH | HIGH | LOW | LOW | LOW |  |
|  |  |  |  |  |  |  |  |  |
|  |  |  |  |  |  |  |  |  |
|  |  |  |  |  |  |  |  |  |
| **Observational Studies** | | | | | | | | |
| **Author** | **Representiveness of the exposed cohort** | **Selection of the non-exposed cohort** | **Ascertainment of exposure** | **Demonstration that outcome of interest was not present at satrt of study** | **Compare ability of cohorts on the basis of the design or analysis** | **Assessment of outcome** | **Was follow up long enough for outcomes to occur** | **Adequacy of follow up of cohorts** |
| Galderisi, M,et al.2009 | NO | NO | YES | YES | YES | YES | YES | YES |
| Eshtehardi, P,et al.2012 | YES | YES | YES | YES | YES | YES | YES | YES |
| Motz, W,et al.1996 | YES | NO | YES | YES | YES | YES | YES | YES |
| Caliskan, M,et al.2007 | YES | YES | YES | YES | YES | YES | YES | YES |
| Galderisi, M,et al.2004 | YES | YES | YES | YES | YES | YES | YES | YES |
| Lethen, H,et al.2011 | YES | YES | YES | YES | YES | YES | YES | YES |
| J-Pmoya, Toms,et al.2006 | YES | YES | YES | YES | YES | YES | YES | YES |
| Sun, B. J,et al.2014 | YES | NO | YES | YES | YES | YES | YES | YES |
| Jensen, L. O,et al.2006 | YES | NO | YES | YES | YES | YES | YES | YES |
| Baller, D,et al.1999 | YES | NO | YES | YES | YES | YES | YES | YES |
| Schwartzkopff, B,et al.2000 | YES | NO | YES | YES | YES | YES | YES | YES |
| Vogt, M,et al.1995 | YES | YES | YES | YES | YES | NO | YES | YES |
| Fujimoto, K,et al.2004 | YES | NO | YES | YES | YES | YES | YES | YES |
| Stamatelopoulos, K,et al.2014 | YES | YES | YES | YES | YES | YES | YES | YES |
| Kjear, A,et al.2009 | YES | YES | YES | YES | YES | YES | YES | YES |
| Kawata,T,et al.2009 | YES | YES | YES | YES | YES | YES | YES | YES |
